# How Fast Can a Human Run? − Bipedal vs. Quadrupedal Running

**DOI:** 10.3389/fbioe.2016.00056

**Published:** 2016-06-30

**Authors:** Ryuta Kinugasa, Yoshiyuki Usami

**Affiliations:** ^1^Department of Human Sciences, Kanagawa University, Yokohama, Kanagawa, Japan; ^2^Institute of Physics, Kanagawa University, Yokohama, Kanagawa, Japan

**Keywords:** 100-m sprint time, quadrupedal running, trend extrapolation, historical race data, biomechanics

## Abstract

Usain Bolt holds the current world record in the 100-m run, with a running time of 9.58 s, and has been described as the best human sprinter in history. However, this raises questions concerning the maximum human running speed, such as “Can the world’s fastest men become faster still?” The correct answer is likely “Yes.” We plotted the historical world records for bipedal and quadrupedal 100-m sprint times according to competition year. These historical records were plotted using several curve-fitting procedures. We found that the projected speeds intersected in 2048, when for the first time, the winning quadrupedal 100-m sprint time could be lower, at 9.276 s, than the winning bipedal time of 9.383 s. Video analysis revealed that in quadrupedal running, humans employed a transverse gallop with a small angular excursion. These results suggest that in the future, the fastest human on the planet might be a quadrupedal runner at the 2048 Olympics. This may be achieved by shifting up to the rotary gallop and taking longer strides with wide sagittal trunk motion.

## Introduction

Currently, Usain Bolt holds the world record in the 100-m run with a running time of 9.58 s. Accordingly, he has been described as the best human sprinter in history. However, this raises questions concerning whether humans can run faster, which means increasing the maximum speed. Over the past 50 years, many researchers have attempted to generate a model to explain the development of world record speeds (e.g., Whipp and Ward, 1992; Tatem et al., 2004; Nevill and Whyte, 2005; Barrow, 2012). One interesting attempt was the calculation of the maximum running speed of Usain Bolt. Using mathematical assumptions, Barrow (2012) predicted that Usain Bolt could achieve a faster running time (from 9.58 to 9.45 s) with no extra effort on his part or improvement in his performance. However, these mathematical models provide no reliable biomechanical and physiological justifications.

Let us consider future athletes. How fast could they run? Although in the past, humans experienced walking and running using two arms and two legs, most humans eventually became bipedal. The existence of quadruped humans (Ledford, 2008; Ozcelik et al., 2008) was first publicized by a 2006 British television documentary about a Turkish family in which several adults walked on all four limbs. In addition to living on all fours, running on all fours has also been reported. One quadruped runner, or “monkey runner,” broke the Guinness world record for the 100-m sprint on November 12, 2015. The new world record time was 15.71 s (Swatman, 2015). Surprisingly, world record times have been set on seven occasions in 7 years and have improved by more than 2 s in the last 4 years. Accordingly, these rapid improvements in quadrupedal running world records might suggest a hypothesis that quadrupedal running will outpace bipedal running in the future.

In this study, to obtain approximate estimates of future 100-m sprint world records, a trend extrapolation method was applied to the world record 100-m bipedal and quadrupedal sprint times by competition year. This study also investigated gait classification and limb kinematics during quadrupedal running, which play a crucial role in aspects such as increasing running speed to certain biomechanical demands such as flexible spine movement. The historical progression of 100-m sprint times and galloping mechanics will likely be important to an understanding of the biomechanical and physiological determinants underlying this seemingly inexorable progression of record performance.

## Methods

### Historical Records Analysis

The bipedal (men’s) and quadrupedal world record 100-m sprint times were obtained from the International Association of Athletics Federations (Hymans and Matrahazi, 2015) and the Guinness World Records (Swatman, 2015) (Table S1 in Supplementary Material). Occasionally, a single individual achieved the world record time in multiple years; however, this analysis assumed that the individual data points were independent.

The historical race data were fitted with use of the Curve Fitting Toolbox (version 3.1.1) of the Matlab software (MathWorks, Natick, MA, USA). Curve-fitting procedures were performed with linear, quadratic, cubic, logarithmic, inverse, power, and exponential curves. Using the same method as that used by Tatem et al. (2004), we used the SE equation to calculate the 95% confidence intervals for the predicted 100-m sprint world records as follows:

$$SE=\sqrt{s_{t,y}^{2}\left(1+\frac{1}{n}+\frac{(y-\overset{\;}{\bar{y}{)}^{2}}}{\sum (y-\overset{\;}{\bar{y}{)}^{2}}}\right)},$$

where *t* is the world record time, *y* is the year, *s* is the sample SD, and *n* is the sample size.

### Movie Analysis

Galloping humans were filmed using a high-speed camera (EXLIM PRO EX-F1, Casio Computer Co., Ltd., Tokyo, Japan) at 300 frames/s. Film recordings were collected during a competitive four-legged sprint race in November 2013. This study was approved by the Kanagawa University Ethics Review Committee for Research Involving Human Participants (2012-1). The analyzed footage of galloping humans also included clips from online resources on YouTube. Galloping cheetahs were analyzed from available free movies of cheetah running (Smith, 2012). In June 2012, the *National Geographic* magazine filmed a cheetah who was radar timed at up to 98 km/h and clocked a time of 5.95 s in a 100-m trial. After a preliminary screening, clips of six different humans and one cheetah were selected. A video clip was selected if it showed a clear sequence of straight linear galloping strides.

Stride-based gait analysis generally considers the contact of the trailing hindfoot as the starting point of the stride cycle (Hildebrand, 1977; Alexander, 2006). In the relative limb phase, the fraction of a stride in which the left forefoot, right hind, and right forefoot touchdown following the left hindfoot touchdown was calculated. It was calculated by counting the frames of the filmed motion sequences based on the method of Hildebrand (1976, 1980). Excursions of the upper arm and thigh segments were thought to be key to achieving a large stride length during quadrupedal running in animals as well as humans, as more distal segments, such as the forearm, hand, lower leg, and foot, play a minor role in excursion. The angular excursion of the upper arm segment was, hence, measured as the angle between the lines connecting the points of the elbow, shoulder, and hip joints. Meanwhile, the angular excursion of the thigh segment was measured as the angle between the lines connecting the points of the shoulder, hip, and knee joints. Stance distance was measured as the linear distance from the left fore to the left hind. Differences in the angular excursion and stance distance among gait cycle were examined with one-way analysis of variance. *Post hoc* comparison (Bonferroni) was performed when significance was found.

## Results

**Figure 1:**
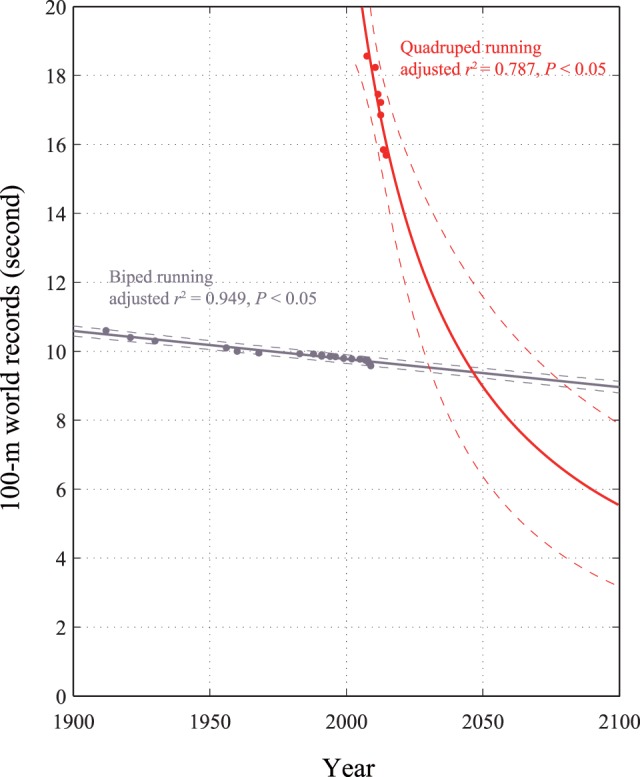
**The 100-m world records for quadrupedal (red points) and bipedal (gray points) human athletes with superimposed best-fit lines and coefficients of determination**. The lines are extrapolated, and the available points are used to superimpose 95% confidence intervals (dotted lines). The projections intersect in 2048, when the quadrupedal 100-m sprint world record will be lower, at 9.276 s, than the bipedal world record of 9.383 s.

We plotted the 100-m sprint times of the bipedal and quadrupedal world records according to competition year. The *r*^2^ values of the quadrupedal times ranged from 0.823 (rational fraction) to 0.968 (cubic), with a mean of 0.846. The *r*^2^ values of the bipedal times ranged from 0.952 (linear) to 0.957 (cubic), with a mean of 0.953. No significant differences in *r*^2^ values were found among the curve-fitting models. The winning time was fitted to a rational fraction curve for the quadruped records (*r*^2^ = 0.823, adjusted *r*^2^ = 0.787, *F* = 26.9, *P* < 0.05) and to a linear curve for the biped records (*r*^2^ = 0.952, adjusted *r*^2^ = 0.949, *F* = 336.1, *P* < 0.05; Figure 1). A rational fraction function can often be used to model complicated structures with a fairly low degree (NIST/SEMATECH e-Handbook of Statistical Methods, 2013). As rational fraction function has excellent extrapolatory powers, it can be favored not only to model the function within the domain of the data but also to be in agreement with theoretical and asymptotic behavior outside the domain of interest. Hence, this study chose the rational fraction curve for the quadruped record extrapolation. The latter process (linear fitting for the bipedal record) was thought to be similar to that described in an earlier literature (Tatem et al., 2004), in which a linear model was used to extrapolate the 100-m sprint time. No indication was found that either bipedal or quadrupedal athletes have reached a plateau in the 100-m sprint. The projected record times intersected in 2048, when for the first time, the projected winning quadrupedal 100-m sprint time was expected to be lower, at 9.276 s, than the bipedal winning time of 9.383 s. By the 2048 Olympic Games, the fastest human on the planet might be a quadrupedal runner, if current trends continue.

**Figure 2:**
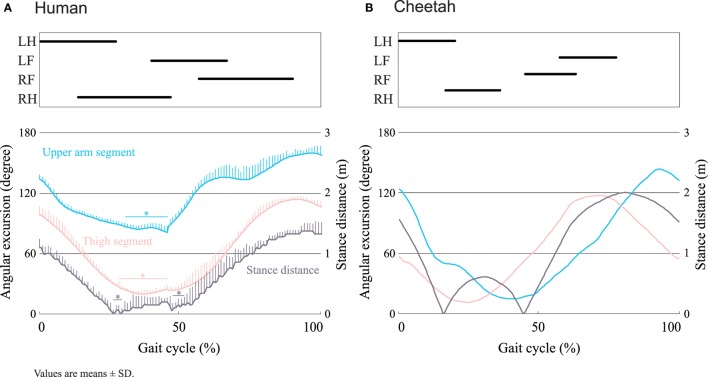
**Diagrams of gait (top illustration), angular excursion, and stance distance (bottom illustration) during quadrupedal running in humans (A) and cheetahs (B)**. On the gait diagram (top illustration), the black thick lines represent the stance periods for each limb during a complete stride. LH, left hind; LF, left fore; RF, right fore; RH, right hind. On the sequential line graph (bottom illustration), the angular excursion of the upper arm (pink) and thigh (blue) segments, and stance distance (gray) are compared over the course of one stride cycle between the humans (*n* = 6) and cheetahs (*n* = 1). *P < 0.05 vs. 0% gait cycle.

Although gallop has been widely considered to be a gait, transverse and rotary gallops can be distinguished. The resulting transverse or rotary gallop depends on whether the two limbs employ the same unilateral or opposite, that is, counter-lateral, skipping gaits according to Minetti (1998). Humans used a transverse gallop, whereas cheetahs used a rotary gallop (Figure 2, top illustration). In humans, the two hindfeet were placed in sequence. Placement of the second hindfoot was followed by placement of the contralateral forefoot, followed by the remaining forefoot. The right–left sequence was the same in the forelimbs and hind limbs. In cheetahs, the placement of the second hindfoot was followed by that of the ipsilateral forefoot, and the sequence of footfalls appeared to rotate around the body. The extended aerial phase duration was also presented. The angular excursion varied between segments and species (Figure 2, bottom illustration). It ranged from 80° to 150° for the upper arm and from 20° to 110° for the thigh in humans and from 10° to 120° for the upper arm and from 10° to 140° for the thigh, in cheetahs. A large angular excursion of the upper arm and thigh segments would yield a great stance distance in cheetahs. The angular excursion at 0% gait cycle was significantly larger than that at 32–46% gait cycle for the upper arm segment and at 30–46% gait cycle for the thigh segment (Figure 2A). The stance distance at 0% gait cycle was significantly longer than that at 27–30% and 48–52% gait cycle (Figure 2A).

## Discussion

It is worth questioning why and how the world records of quadrupedal human athletes are expected to improve more rapidly than those of bipedal human athletes (Figure 1). To answer this question, we used video analysis to demonstrate the gait classification and kinematic pattern during quadrupedal running. The first potential answer is gallop. Many mammals employ asymmetrical gaits, such as the two forms of gallop, known as transverse and rotary, particularly at high speeds. Our study showed that quadrupedal running humans employed transverse gallop (Figure 2). Horses are known to only perform transverse gallop at any speed, while cheetahs and racing dogs have clearly been known to perform the rotary gallop (Hildebrand, 1977). A kind of continuous gradient from transverse to speed-dependent to rotary species such as that in some dogs (Walter and Carrier, 2007) is quite evident (Gambaryan, 1974; Hildebrand, 1977; Bertram and Gutmann, 2009). These observations indicate that a transition from transverse to rotary gallop may achieve a higher speed in quadrupedal running humans.

The second potential answer is trunk movement. Sagittal spine movements in mammals are important during asymmetrical gaits, particularly in small mammals and larger cursorial species, wherein cyclic flexion and extension of the spine help to increase the stride length (Hildebrand, 1959; Schilling and Hackert, 2006). In a different perspective, on the robotic platforms, flexible spine movement also allows an increase in the maximum running speed (Culha and Saranli, 2011; Moore et al., 2015). In galloping, faster speeds are achieved by taking longer strides, although the stride frequency remains almost constant (Biewener, 2003). Thus, longer legs and a large limb excursion represent features that are important to achieving faster running speeds. Fortunately, in human quadrupeds, the distal segment of the leg usually lengthens more than the proximal segment (Winter, 1990), yielding not only longer legs but also a longer moment arm for the distal segment. Thus, relatively larger ground forces can be applied, which reduce the limb contact time and increase the flight time (Weyand et al., 2000). Our kinematic analysis indicated that small angular excursion was observed in humans rather than in cheetahs (Figure 2), which means that human spine dorsoventral and lateral stiffness are largely limited during quadrupedal running. The reason for the inflexibility may, in part, be due to a long, robust, and stiff thoracic region, a stiff lumber spine of variable length, and little mobility at the lumbosacral joint [e.g., Galis et al. (2014)]. In humans, however, an increase in sagittal spinal movement might lead to an extended stride, an adaptation that could allow for higher speeds.

This study has limitations. Although statistical models are significantly related to mathematical formula, the use of a statistical model to accurately predict future athletic performance is challenging (Hilbe, 2008). Fitted linear models should be treated with some caution. The use of linear regression for world record modeling would yield a continued decline that would eventually become negative, thus suggesting that update of world records can be continued until 0 s. It must also be noted that quadrupedal world records did not exist before 2008. This relatively recent involvement of quadrupedal running results in a somewhat tenuous comparison of world record times. Therefore, despite a high coefficient of determination, a large diverging confidence interval was found. The 95% confidence intervals indicates that projected intersects could occur as early as in 2032 (9.238 s) or as late as 2076 (9.341 s). In the future, the number of data should be increased for a conclusive answer of quadrupedal world records.

In summary, in the future, the fastest humans on the planet might be a quadrupedal runner at the 2048 Olympics, which may be achieved by shifting up to the rotary gallop and taking longer strides with wide sagittal trunk motion. More generally, investigation of quadrupedal running will not only result in the development of new techniques that allow biomechanists to study locomotion in natural settings but will also reveal the underlying principles of how these runners accomplish their astonishing performances; this may also result in the development of novel actuation materials that are lightweight, robust, compliant, and easy to manufacture into various designs.

## Author Contributions

RK made substantial contributions to the conception and design of the work. RK and YU contributed toward the acquisition, analysis, and interpretation of data for the work. Drafting the work and revising it critically for important intellectual content was performed by RK and YU. RK and YU approved the final version to be published.

## Conflict of Interest Statement

The authors declare that the research was conducted in the absence of any commercial or financial relationships that could be construed as a potential conflict of interest.
