# Supplementary material for: How Fast Can a Human Run? − Bipedal vs. Quadrupedal Running
Source: Front Bioeng Biotechnol. 2016 Jun 30;4:56. doi: 10.3389/fbioe.2016.00056 (PMC4928019; doi:10.3389/fbioe.2016.00056)
Supplement: Supplementary file 1 [file table_1.pdf]

Table S1. Bipedal and quadrupedal world record 100-m sprint times from 1912.

Year	100-m world records (s)	
	Bipedal[1)]	Quadrupedal
1912	10.6	
1921	10.4	
1930	10.3	
1956	10.1	
1960	10.0	
1968	9.95	
1983	9.93	
1988	9.92	
1991	9.90	
1991	9.86	
1994	9.85	
1996	9.84	
1999	9.79	
2002	9.78	
2005	9.77	
2007	9.74	
2008	9.72	18.58
2008	9.69	
2009	9.58	
2011		18.25
2012		17.47
2013		17.23
2013		16.87
2014		15.86
2015		15.71[2)]

Source: [1)] International Association of Athletics Federations (Hymans and Matrahazi 2015).

[2)] Guiness World Records. Watch Japan’s Kenichi Ito scamper to GWR Day success with fastest 100 m running on all fours (Swatman, 2015).
